# Air pollution, investor sentiment and stock liquidity

**DOI:** 10.3389/fpubh.2022.989457

**Published:** 2022-10-06

**Authors:** Chenggang Li, Ying Yan, Yi Lu, Guifeng Zeng, Liying Zhou, Han Jin, Yunbao Xu, Yuzhu Chen

**Affiliations:** ^1^School of Big Data Application and Economics, Guizhou University of Finance and Economics, Guiyang, China; ^2^Green Development Strategy Research Institute in Western China, Guizhou University of Finance and Economics (First Batch of New Characteristic Think Tanks in Guizhou), Guiyang, China; ^3^Business School, Sichuan University, Chengdu, China; ^4^Archives of Hunan Institute of Engineering, Xiangtan, China; ^5^Business School, Guizhou University of Finance and Economics, Guiyang, China; ^6^Guizhou Key Laboratory of Big Data Statistical Analysis, Guizhou University of Finance and Economics, Guiyang, China; ^7^School of Economics, Hunan Institute of Engineering, Xiangtan, China; ^8^School of Foreign Languages, Guizhou University of Finance and Economics, Guiyang, China

**Keywords:** air pollution, air quality index, investor sentiment, stock liquidity, turnover rate

## Abstract

With the aggravation of air pollution, the impact of air pollution on the stock market, especially from the perspective of investor sentiment, has been of great concern and widely discussed. Based on data from China's A-share listed firms from January 1, 2016, to December 31, 2020, the relationship between urban air pollution and stock liquidity of listed firms and the internal mechanism is examined. Firstly, based on local preference theory, we start by predicting the impact of air pollution on stock liquidity. We, then, build a regression model for air pollution and stock liquidity, introducing the intermediary effect model to detect the relationship between the two and its mechanism. Finally, by subdividing the samples, we discuss the differential impact of air pollution on stock liquidity under different circumstances. We found that when air pollution worsens it reduces stock liquidity. The results of the mechanism analysis show that investor sentiment plays an intermediary role in the process of air pollution affecting stock liquidity, and pessimism induced by air pollution can reduce stock liquidity. Heterogeneity test results show that there are differences in the impact of air pollution on stock liquidity between heavily polluting firms and non-heavily polluting firms, different industries, different city sizes, and different levels of air pollution, has a greater effect in non-heavily polluting enterprises, manufacturing and other industries, medium sized cities and light pollution. The results of this research have important reference value for environmental protection departments to establish and improve air pollution monitoring systems and for listed firms to improve stock liquidity and deal with the environmental financial risks appropriately.

## Introduction

The 18th National Congress of the Communist Party of China proposed to vigorously promote the construction of ecological civilization. This strategic decision assists the building of beautiful China and ensures the Chinese nation's sustainable and healthy development. It also reflects China's firm belief in taking the initiative to lead the development trend for human civilization. In recent years, China has successively implemented three major action plans for the prevention and control of air, water and soil pollution, continuously strengthened the control of environmental pollution, improved the level of air pollution supervision, and continuously improved the ecological environment (1). At the same time, the problem of air pollution is getting more and more attention. Numerous studies have shown that air pollution can lead to negative psychological and physiological reactions. For example, air pollution can lead to increased incidents of respiratory disease, cardiovascular disease, and increased use of emergency rooms (2–5). Landrigan (6) identified air pollution as a major yet under-recognized cause of non-communicable diseases. Glencross et al. (7) demonstrated the association of known elevated environmental pollution with asthma and chronic obstructive pulmonary diseases. There is no doubt that air pollution damages physical health. However, whether air pollution affects people's mood and thus stock market liquidity has not received sufficient attention.

Liquidity is the vitality of the securities market, and is an important indicator of whether the stock market is functioning well. Good liquidity not only helps to improve the efficiency of resource allocation, but also guarantees the normal operation of the stock market (8, 9). The deterioration of stock liquidity means more stringent trading conditions in the secondary market, resulting in only a fraction of stocks able to be successfully traded, which leads to a loss in the attractiveness of the stock market. In addition, illiquid markets are often prone to liquidity crises, for example, the European monetary system crisis in 1992, the Mexican peso crisis in 1994, the Southeast Asian financial crisis in 1997, the U.S. subprime mortgage crisis in 2008, the 1,000 share crash in China's A-shares in June 2015, and the four liquidity meltdowns in the U.S. market on March 19, 2020, all resulted from deteriorating stock liquidity (10). The importance of maintaining good stock liquidity cannot be overstated. Moreover, in terms of liquidity costs, although China's stock market liquidity has improved significantly in the last decade, there is still a gap with international markets. Therefore, the study of stock liquidity is of great practical importance.

Air pollution has been shown to have an impact on stock performance, and the vast majority of studies have concluded that air pollution significantly reduces stock market returns. On one hand, air pollution in stock exchange locations is negatively associated with market index returns (11), on the other hand, the stock liquidity of firms located in heavily polluted cities is more vulnerable to air pollution (12–14). Academic opinions on the impact of air pollution on the stock market can be summarized as follows: One view is that air pollution acts on the stock market by affecting the emotions of stock exchange traders (15). Another view is that air pollution negatively affects investors' emotions, changes investors' propensity to trade or reduces trading frequency, and affects stock liquidity (14). This view can also be explained by psychological theories, with the psychological literature founding that environmental factors affect emotions, and that emotions influence thinking, judgment, and decision making (16, 17). Specifically, in complex decision situations involving risk and uncertainty, investors in negative states of mind are more pessimistic about stock pricing than investors in a neutral state of mind, while investors in a positive state of mind are more optimistic about stock pricing. Given that social interactions are an important aspect of the decision-making process, society's general optimism or pessimism is transmitted through such interactions and influences the decisions of all investors, including stock investments (18). When in an environment of severe air pollution, investors are prone to low mood and are more pessimistic about stock pricing, raising their propensity to sell stocks.

Although some scholars have proved that air pollution can affect stock return, few studies have explored the impact of air pollution on stock liquidity. Although some studies discuss the relationship of air pollution and stock liquidity, they have not clarified the impact mechanism of air pollution on stock liquidity. We use the data of China from 2016 to 2020 to explore the following issues: whether the air pollution affects the stock liquidity, how the air pollution affects the stock liquidity, and whether the relationship between air pollution and stock liquidity changes under different circumstances. This study contributes to the existing literature in a number of ways. Firstly, based on the micro perspective, we expands the existing research on the economic consequences of air pollution, and further provides evidence for the impact of air pollution on the stock market from the perspective of behavioral finance. Secondly, it not only enriches research on the influencing factors of stock liquidity, but also provides decision-making reference for enterprises on how to better deal with and resolve stock liquidity risks, and provides basis for the government to implement differentiation policies. Thirdly, this study has made contributions to public health and environmental health. This study will enable the public to understand the causes of emotional changes and increase their attention to air pollution, and take timely measures to regulate psychological emotions, prevent and reduce the impact of air pollution on people's physical health. This study provides a new empirical evidence for the role of air pollution, public health and the stock market. The financial market is crucial to a country's economic development, which shows that air pollution is not only an important social problem, but also an economic problem. Whether from a social or economic point of view, government departments will consider strengthening the prevention and control of environmental pollution, strive to improve environmental pollution to the greatest extent. In the rest of the paper Section Air pollution and stock liquidity in China discusses air pollution and stock liquidity in China, Section Methodology contains the methodology and data, Section Results and discussion gives the results and discussion, and Section Conclusion and implications presents the conclusion and implications of study.

## Air pollution and stock liquidity in China

**Figure 1:**
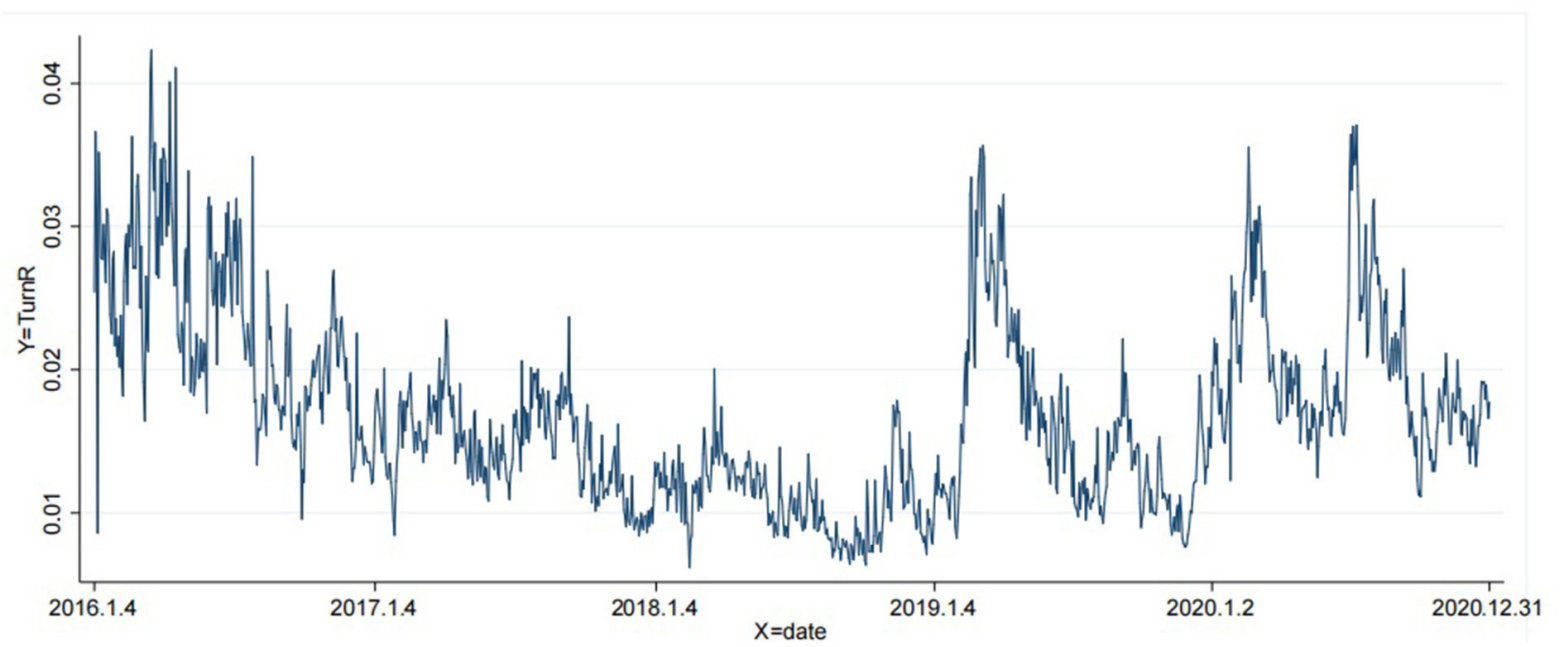
Stock liquidity trends from 2016 to 2020.

Figure 1 shows the trend of stock liquidity of sample companies from 2016 to 2020. As seen in Figure 1, on the whole, the liquidity of the sample companies in each period has numerous differences and a large fluctuation range, and this increases greatly at the beginning of each year, but it is still at a low level. At the beginning of 2016, stock liquidity gradually declined and became worse. Stock liquidity showed a downward trend from 2016 to 2018. Equity liquidity has improved since the beginning of 2019, with less volatility in equity liquidity in 2020.

**Figure 2:**
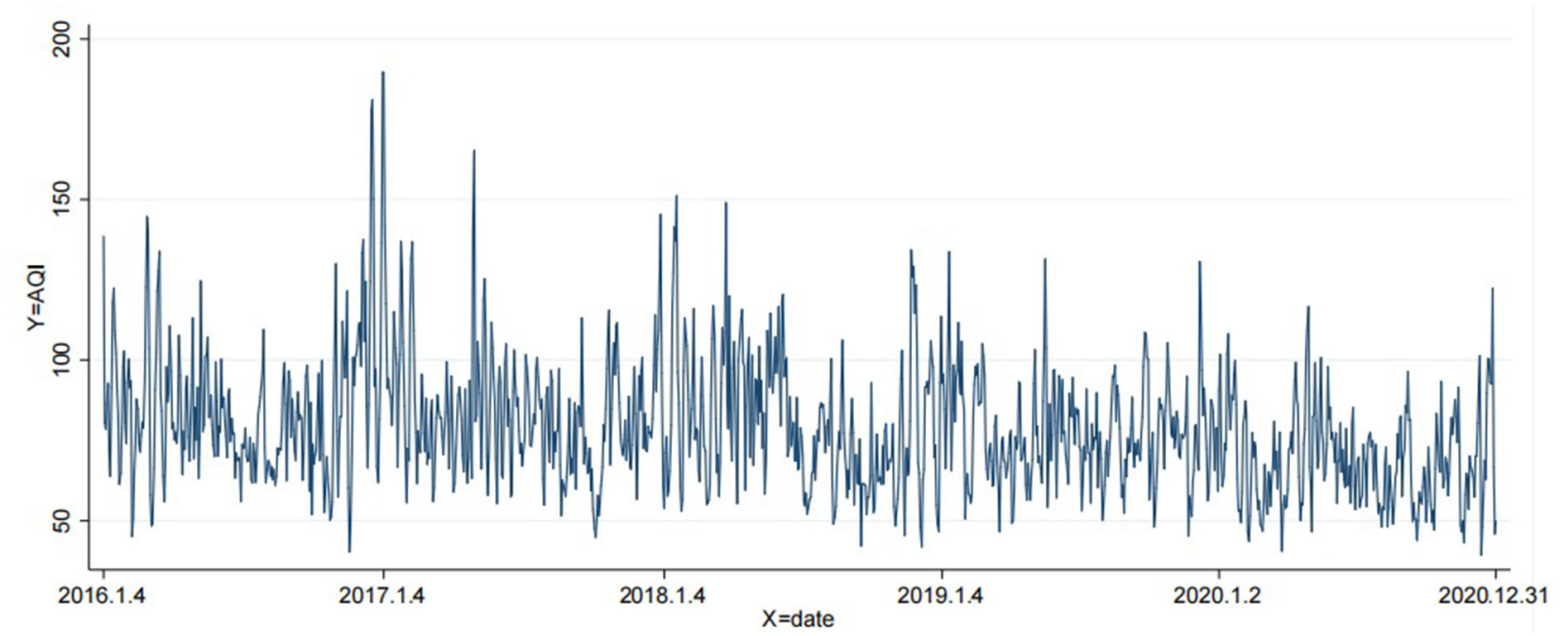
Air pollution trends from 2016 to 2020.

Figure 2 shows the change trend of air pollution from 2016 to 2020. According to Figure 2, generally speaking, there is a large gap in air pollution during the year, and the air pollution at the beginning of each year is relatively serious, at a high level.

**Figure 3:**
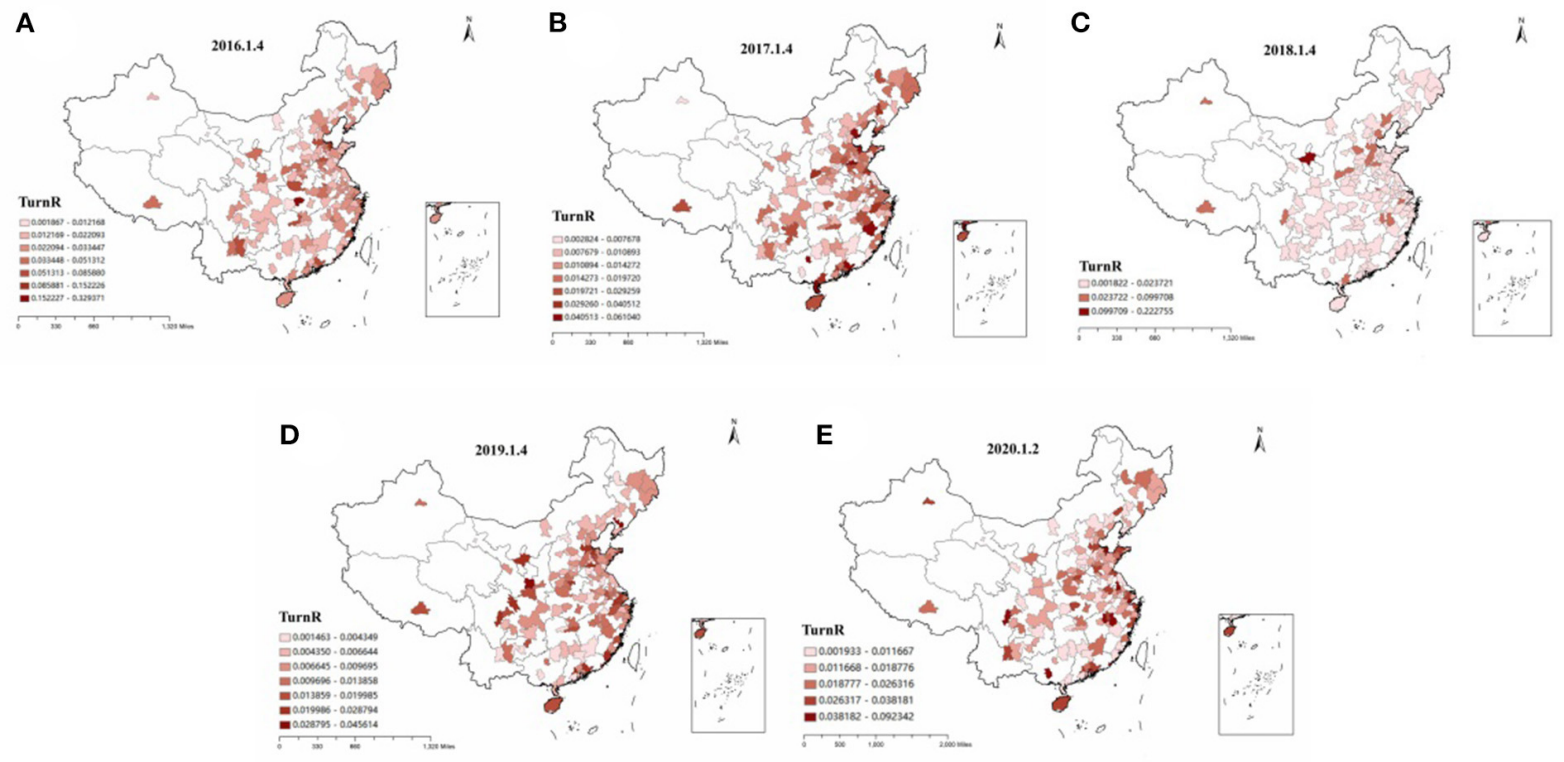
**(A–E)** Spatio-temporal evolution of corporate stock liquidity in different regions of China.

Figure 3 describes the evolution of stock liquidity of listed companies in various prefecture-level cities in China over time. Generally speaking, the stock liquidity of the enterprises has improved during this period. The stock liquidity of the companies in the central region at the beginning of 2016 and 2017 was at a low level, and the stock liquidity of most regions in 2018 was better. In 2019, the stock liquidity of enterprises located in Shandong Province and the southern part of Shanxi Province was at a low and medium level. In 2020, the stock liquidity of enterprises in various regions was significantly improved.

**Figure 4:**
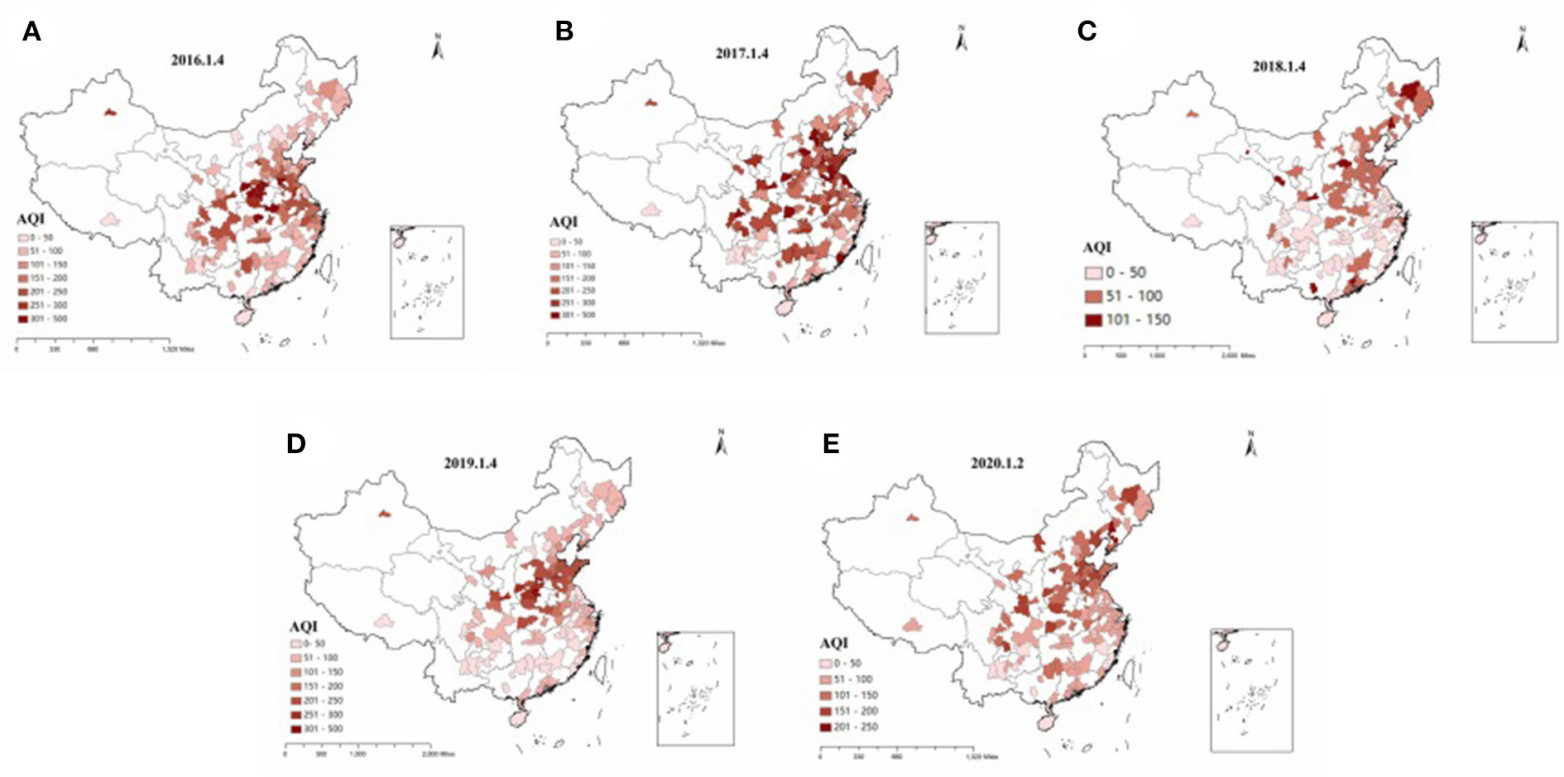
**(A–E)** Spatio-temporal evolution of air pollution in different regions of China.

Figure 4 describes the evolution of air pollution conditions in various prefecture-level cities in China over time. According to Figure 4, on the whole, air pollution in sample cities improved significantly from 2016 to 2020. In 2016 and early 2017, there were still serious pollution areas (AQI>300), concentrated in central China. In 2018, the air quality was good, and there were no seriously polluted areas. The AQI of most prefecture-level cities was in the range of 0–150, showing mild pollution. In 2019, a small number of prefecture-level cities were seriously polluted, mainly in southern Shanxi and Shandong provinces. In 2020, air pollution in prefecture-level cities improved, with no severely polluted areas. Compared with 2019, the range of severely polluted areas (201<AQI<300) was significantly reduced. Comparing Figures 3, 4, we can find that the areas with serious air pollution are generally consistent with the areas with low stock liquidity.

## Methodology

### Model

In order to investigate the relationship between urban air pollution status and stock liquidity of listed firms, We constructed the following regression model.


(1) $$\begin{array}{l} TurnR_{i,t,d}\;=\;\alpha +\beta_{0}AQI_{i,d,c}+\beta_{1}CSI_{d}+\beta_{2}ARED_{i}+\gamma EXD\;\\ \;+\;\eta_{1}Monday+\lambda_{1}Month+Year+City+\phi \\ \;+\;\varepsilon_{i,t,d} \end{array}$$

Where, TurnR_i,t,d_ is dependent variable, representing the stock turnover rate of firm i on day d of year t, α is a constant term, the core explanatory variable AQI_i,d,c_ is the air quality index of city c where firm i is located on day d, which is used to measure the local air pollution condition, and β_0_ is its regression coefficient. The control variable CSI_d_ represents the return of the CSI 300 index on day d. ARED_i_ is the annual report announcement date of listed firm i. The variable is set to 1 if the day is the annual report announcement date, and 0 otherwise. EXD_i_ is the ex-dividend and ex-rights date of listed firm i. The variable is set to 1 if the day is the ex-dividend and ex-rights date, and 0 otherwise, and γ is its effect coefficient. In addition, Monday represents the Monday effect, Month is the month effect, Year, City, and Φ_i_ represent year, city, and firm fixed effects, respectively, and ε_i,t,d_ is the random disturbance term. This model focuses on the coefficient β_0_ of AQI_i,d,c_.

In this study, we select five control variables: Daily return of CSI 300 index (CSI), Annual Report Announcement Date (ARED), Ex-dividend and ex-rights Date (EXD), Monday Effect and Month Effect. The coefficient of CSI, ARED, Monday Effect and Month Effect are expected to be positive. However, the coefficient of EXD is expected to be negative. The reasons are as follows, the overall market characteristics, such as the market rate of return, will affect the level of stock liquidity (19). The daily rate of return of the Shanghai and Shenzhen 300 index is selected to represent the market rate of return. The research results of Chen et al. (20) show that the trading volume on the day of publication of the annual report is 1.75 times of the average annual trading volume. Chen and Chen (21) also found that when the company announced its annual report, the stock trading volume increase significantly. In order to obtain dividends or reduce tax burden, investors will choose to conduct stock trading activities before or after the ex-dividend date (22). In addition, due to the cumulative effect of weekend information, there are more informed traders and private information in the market on Monday (23). On Monday, investors will conduct stock trading whether they guess the good news and gain profits, or when there is bad news and loss or news vacuum (24). Fan and Dong (25) found that Chinese investors tend to chase stocks with good market performance in March, and tend to get rid of stocks with poor market performance in December, which will increase the trading frequency in March and December.

The turnover rate can be obtained by Equation (2), given below.


(2) $$\begin{array}{l} TurnR_{itd}=\frac{VOL_{itd}}{LNS_{itd}}*100 \end{array}$$

where TurnR_itd_ is the turnover rate of stock i on day d of year t, representing the stock liquidity on that day, VOL_itd_ is the number of shares traded in stock i on day d of year t, and LNS_itd_ is the number of shares outstanding in stock i on day d of year t. The larger the value of the turnover ratio, the more frequently a stock changes hands, and the more liquid the stock is. Smaller values of the turnover ratio are multiplied by 100 for ease of measurement.

In order to explore the path of its action mechanism, the mediating effect model was constructed to test whether investor sentiment is an intermediate bridge for air pollution to affect stock liquidity. The mediation effect is composed of model (3) to model (5).


(3) $$\begin{array}{l} TurnR_{i,t,d}\;=\;\alpha +\beta_{0}AQI_{i,d,c}+\beta_{1}CSI_{d}+\beta_{2}ARED_{i}+\gamma EXD_{i}\\ \;\begin{array}{l} +\;\eta_{1}Monday+\lambda_{1}Month+Year+City+\phi_{i}\\ +\;\varepsilon_{i,t,d} \end{array}\; \end{array}$$

(4) $$\begin{array}{ll} InvS_{d}\;=\;\alpha_{1}+\beta_{3}AQI_{i,d,c}+\beta_{4}CSI_{d}+\beta_{5}ARED_{i}+\gamma EXD_{i}\\ \; & \begin{array}{l} +\;\eta_{2}Monday+\lambda_{2}Month+Year+City+\phi_{i}\\ +\;\varepsilon_{i,t,d} \end{array}\; \end{array}$$

(5) $$\begin{array}{l} TurnR_{i,t,d}\;=\;\alpha_{2}+\beta_{6}AQI_{i,d,c}+\beta_{7}InvS_{d}+\beta_{8}CSI_{d}\\ \;\begin{array}{l} +\;\beta_{9}ARED_{i}+\gamma EXD_{i}+\eta_{3}Monday\\ +\;\lambda_{3}Month+Year+City+\phi_{i}+\varepsilon_{i,t,d} \end{array} \end{array}$$

where TurnR_i,t,d_ is the turnover rate of firm i on day d in year t, AQI_i,d,c_ is the air quality index of city c where firm i is located on day d. As a proxy variable for air pollution, InvS_d_ represents investor sentiment on day d, which is measured by choosing the Advance Decline Line index (hereinafter referred to as “ADL”), Arms index (also known as TRIN, Short-term Trading Index, hereinafter referred to as “ARMS”), and Guba comment sentiment (Sent). The meanings of other variables remain unchanged. The investor sentiment of Guba can be obtained by Equation (6), given below.


(6) $$Sent_{i,t,d}=\text{ln}\left(1+\sum_{d=1}^{D}N_{post,itd}/1+\sum_{d=1}^{D}N_{neg,itd}\right)$$

Where: N_post, itd_ is the number of positive sentiment posts appearing in the Eastmoney Guba on day d of stock i in year t; N_neg, itd_ is the number of negative sentiment posts appearing in the Eastmoney Guba on day d of stock i in year t.

### Hypothesis

**H1: Air pollution will have a negative impact on stock liquidity**

Air pollution has always been the research hotspot all over the world. Many studies in this area show its importance. In recent years, the impact of air pollution on stocks has also been a major concern. Some empirical studies discuss the relationship between air pollution and stock liquidity. Wu et al. (14) suggest that severe air pollution reduces local stock returns, liquidity and volatility. The air pollution effect is more pronounced for stocks that are difficult to value and arbitrage, and air pollution also reduces firm-level liquidity. Li and Wang (26) state that air quality index is not significantly associated with stock returns, but has an effect on turnover rates. We apply the fixed effect model to predict the relationship between two variables of interest.

**H1: Changes in investor sentiment caused by air pollution can reduce stock flows. The more severe air pollution is, the more negative investor sentiment is, and stock liquidity becomes less liquid**

The most important characteristic of investors as economic actors in the securities market is their investment decisions (27). According to the local preference theory, in China's capital market, investors' familiarity with and overconfidence in local stocks will lead to behavioral bias, and they choose to buy more shares of local companies when allocating funds (28). It is widely accepted in academia that air pollution acts on stock markets by altering investor sentiment. Levy and Yagil (11) argue that air pollution leads to negative investor sentiment and risk-averse behavior, resulting in a negative correlation between air pollution and stock returns. Zhang et al. (12) found that hazy weather acts on the stock market through investor sentiment in the market. Xu (13) points out that air pollution impairs investor sentiment and increases risk aversion, leading to a decline in stock prices. Li and Wang (26) argue that air pollution acts on stock returns mainly through altering investors' sentiment and authorities' policymaking, which leads to stock market volatility. Wu et al. (14) suggest that air pollution is one of the factors contributing to pessimism and that severe air pollution reduces local stock returns, liquidity, and volatility. Shi and Guo (29) examine the effect of haze pollution on stock performance, showing that haze pollution has a significant negative effect on stock returns and a significant positive effect on stock volatility, where the influence channel is investor sentiment. We test this hypothesis by applying the mediation effect model.

**H1: The impact of air pollution on stock liquidity is more significant in heavy polluting enterprises, manufacturing and other industries, large cities and light air pollution**

Air pollution forces the government to introduce some environmental policies, which affects investors' strategies, especially in China's A-share market (30). Compared with other industries, heavily polluting enterprises and manufacturing industries face stricter external supervision and policy constraints. In this case, the impact of air pollution on stock liquidity change. In addition, big cities with large populations and factories have worse air quality, all things being equal. Studies have shown that different degrees of air pollution have different effects on emotions (31). Therefore, we consider using grouping experiments to test the different effects of air pollution on stock liquidity under different conditions.

### Data

We selected Chinese A-share listed companies from January 1, 2016 to December 31, 2020 as the research sample, and the data involved included air pollution, stock liquidity index, return rate of Shanghai Shenzhen 300 index [the CSI 300 Index), annual report announcement days, ex-dividend and ex-right dates. The data of stock liquidity, CSI 300 index return, annual report release date and ex-dividend and ex-right dates are from CSMAR database and RESSET database. The air pollution data are from the official website of China Meteorological Bureau. Excluding ST, ^*^ST companies and companies with serious data deficiency, we finally get 853437 observations. Definitions of variables and descriptive statistics of variables are given in Table 1.

**Table 1:** Variable definition and descriptive statistics.

**Variables**	**Number**	**Minimum**	**Maximum**	**Average**	**Std. dev**.	**Definition**
**Descriptive statistics of continuous variables**						
Turnover rate	853,437	0.0020	70.9508	1.6991	2.4743	The frequency with which stocks change hands in the market during a certain period of time.
AQI	853,437	12	500	79.0079	42.5131	AQI describes how clean or polluted the air is and measures the air pollution in a city over a period of time
CSI	853,437	−0.0821	0.0578	0.0002	0.0126	Daily return of CSI 300 index
ADL	853,437	−2668	2806	−7.2002	1291.885	ADL is used to measure investor sentiment
ARMS	853,437	0.1957	5.9168	0.9306	0.4593	ARMS is used to measure investor sentiment
Guba posting sentiment	848,576	−4.0431	5.1762	0.2586	0.7522	Eastmoney Guba posting sentiment, used to measure investor sentiment
Amihud	853,437	0	479.8357	0.0331	0.5449	Amihud is an inverse measure of stock liquidity
		**Vaule** = **1**		**Vaule** = **0**		
**Variables**	**Total number**	**Number**	**%**	**Number**	**%**	**Definition**
**Descriptive statistics of categorical variables**						
Annual report announcement date	853,437	2,209	0.26%	851,228	99.74%	Dummy variable, set to one on the day of annual report announcement, zero otherwise
Ex–dividend and ex–rights date	853,437	2,480	0.29%	850,957	99.71%	Dummy variable, set to one on ex–dividend and ex–rights day, zero otherwise
Month effect	853,437	153,865	18.03%	699,572	81.97%	Dummy variable, set to one when month is March or December, zero otherwise
Monday effect	853,437	167,216	19.59%	686,221	80.41%	Dummy variable, date set to one for Monday, zero otherwise

In this study, the air quality index is used to measure air pollution. Turnover rate is used to measure stock liquidity. We use other determinants as control variables, such as the daily yield of the CSI 300 index, the announcement date of the annual report, the ex-dividend and ex-right dates, the Monday effect and the month effect, and add the year, city and enterprise dummy variables. ADL index and ARMS index are used to measure investor sentiment. The ADL indicator is the increase minus the decrease of the stock market on a given day. The ARMS indicator indicates the proportional relationship between the number of rising and falling stocks. In addition, in the participation and communication of the stock forum, people tend to choose geographical location to participate in specific discussion topics, and investors are more likely to participate in the exchange of local stock information (32). The sentiment of the stock forum to a large extent represents the sentiment of local investors. Therefore, we also regard stock bar comment sentiment as an indicator of investor sentiment. The analysis data was collected from the CSMAR database, the China Meteorological Network and the CNRDS database.

## Results and discussion

This section introduces the results of the regression model. Table 2 shows the benchmark regression results. Tables 3–6 show the results based on the impact of air pollution on stock liquidity under different circumstances. Table 7 shows how air pollution affects stock liquidity.

**Table 2:** Baseline regression of turnover rate and air pollution.

	**(1)**	**(2)**	**(3)**	**(4)**
**Variables**	**Turnover rate**	**Turnover rate**	**Turnover rate**	**Turnover rate**
AQI	−0.0003***	−0.0004***	−0.0006***	−0.0004***
	(6.01e−05)	(6.01e−05)	(6.06e−05)	(6.01e−05)
CSI	–	1.210***	1.845***	1.210***
		(0.1870)	(0.190)	(0.1870)
Annual report announcement date	–	0.555***	0.399***	0.555***
		(0.0462)	(0.0470)	(0.0462)
Ex–dividend and ex–rights date	–	−0.0411	−0.199***	−0.0411
		(0.0436)	(0.0444)	(0.0436)
Monday effect	–	0.0159***	0.0218***	0.0159***
		(0.0059)	(0.0060)	(0.0059)
Month effect	–	0.0668***	0.0787***	0.0668***
		(0.0061)	(0.0062)	(0.0061)
Constant term	2.337***	2.324***	1.725***	2.324***
	(0.0073)	(0.0074)	(0.0056)	(0.0074)
Year effect	Yes	Yes	No	Yes
City effect	Yes	No	Yes	Yes
Individual effects	Yes	Yes	Yes	Yes
Observations	853,437	853,437	853,437	853,437

*** shows significance at 1%; Standard errors are in parentheses.

### Benchmark regression

Table 2 presents the regression results between the stock turnover rate and the AQI of the city where it is located. Column (1) of Table 2 does not include control variables, but includes annual fixed effect, urban fixed effect and individual fixed effect. Column (2) includes all control variables, but includes annual fixed effect and individual fixed effect, and does not control urban fixed effect. Column (3) adds all control variables, but adds urban fixed effect and individual fixed effect, and does not control annual fixed effect. Column (4) adds all control variables and controls annual fixed effect, urban fixed effect and individual fixed effect. In Columns (1) to (4), we can see that the negative impact of the air quality index on the turnover rate of individual stocks is significant at the level of 1%, which means that air pollution has significantly reduced the stock liquidity of listed companies. In Column (4), the coefficient of AQI is −0.0004, which is significant at the significance level of 1%, which indicates that the turnover rate of individual shares of listed companies will decrease by 0.0004 units for each unit of AQI increase. In other words, the aggravation of air pollution will lead to the deterioration of stock liquidity of listed companies. A possible reason for this phenomenon is that investors exposed to air pollution tend to be depressed, and they are more pessimistic about stock pricing, and risk aversion and unwillingness to trade stocks are higher with more serious air pollution (33–36). This, results in lower stock trading frequency, with poor stock liquidity (37). The daily yield of the CSI 300 index is significantly positively correlated with the turnover rate. The coefficient of CSI is 1.210, which means that the stock liquidity of listed companies will increase by 1.210 units for every unit of CSI increase. The possible reason for this result is: when the daily yield of the CSI 300 index is at a high level, it represents an upward market trend, investors have a higher buying tendency and stock liquidity is improved. The coefficient of the annual report release date is 0.555, which is significantly positive at the level of 1%. The annual report announcement is positively related to the turnover rate. This indicates that the stock liquidity will be improved on the day when the listed company releases the annual report. The reason is that investors understand the annual operating conditions of listed companies on the announcement date of the annual report, and will react by buying or selling stocks in the short term, resulting in changes in the liquidity of individual stocks. At the same time, The Monday effect coefficient is 0.0159, which is positive at the level of 1%, indicating that the Monday effect is positively related to the stock turnover rate, that is, compared with other working days, the company's stock trading frequency is higher on Monday. The possible reason for this phenomenon is: investors will make stock trading decisions more frequently on Monday after two days of paying attention to and analyzing the impact of weekend market news on the stock price of listed companies, resulting in changes in the liquidity of individual stocks. The coefficient of the month effect is 0.0668, which is significantly positive at the level of 1%, indicating that the month effect is positively related to the stock liquidity. Compared with other months, the stock liquidity of listed companies is better in March and December, indicating that investors will trade stocks more frequently at the beginning of the spring and at the end of the year.

### Heavily polluting and non-heavily polluting firms

As the public and government pay closer attention to air pollution, environmental regulations, and industrial policy adjustments, some investors may prefer to invest in environmentally friendly firms and sell off the stocks of heavily polluting firms. Therefore, we considers the existence of heterogeneity between heavily polluting and non-heavily polluting firms. According to the catalog of classified management of environmental protection verification industry of listed enterprises (HBH [2008] No. 373) published by the Ministry of Environmental Protection of China in 2008, heavily polluting industries are merged into the following eight categories: mining industry; textile, clothing, leather and wool industry; metal smelting industry; petrochemical and plastic industry; food and beverage industry; hydropower and gas industry; biomedicine industry; paper and printing industry. Using the above classification standards, we divide the sample companies into heavily polluting and non-heavily polluting companies according to their main businesses.

Table 3 presents the regression results for the heavily polluting and non-heavily polluting firms in Columns (1) and (2), respectively. The regression coefficients of the air quality index are significantly negative at the 1% and 5% levels, respectively. The coefficient of AQI is−0.0005 in non-heavy pollution companies and−0.0002 in heavily polluting companies. The estimate of AQI on stock turnover rate for non-heavily polluting firms was higher than that of heavily polluting firms. According to the judgment method proposed by Schenker and gentleman (38), in non-heavily polluting companies, air pollution has a greater impact on stock liquidity.

**Table 3:** Heavily polluting and non–heavily polluting firms.

**Variables**	**Non–heavily**	**Heavy**
	**polluting firms**	**polluting firms**
AQI	−0.0005***	−0.0002**
	(8.05e−05)	(8.99e−05)
CSI	1.7310***	0.4630*
	(0.251)	(0.2800)
Annual report announcement date	0.5290***	0.5910***
	(0.0623)	(0.0682)
Ex–dividend and ex–rights date	−0.0560	−0.0177
	(0.0586)	(0.0647)
Monday effect	0.0059	0.0302***
	(0.0080)	(0.0088)
Month effect	0.0975***	0.0227**
	(0.0082)	(0.0091)
Constant term	2.3700***	2.2590***
	(0.0100)	(0.0111)
Year effect	Yes	Yes
City effect	Yes	Yes
Individual effects	Yes	Yes
Observations	503,051	350,386

***, * *, * show significance at 1%, 5%, and 10% respectively; Standard errors are in parentheses.

Possible reasons for this are that with the increase in air pollution, people began to pay more attention to stocks of environmental protection firms and less attention to firms causing heavy pollution. Coupled with the introduction of relevant national environmental policies, investors are optimistic about the prospects of environmental protection firms, tending to sell stocks of polluting firms and hold stocks of environment protection firms. Investors who are not originally negative about, or less likely to trade stocks of heavy polluters will more often decide to trade stocks of non-heavy polluters rather than heavy polluters, even though they could be influenced in their decision making by the negative sentiment brought by air pollution. Investors mostly face the problem of making stock investment decisions in non-heavily polluting firms, which leads to a greater decline in the frequency of stock trading in non-heavily polluting firms than in heavily polluting firms. Thus, compared with heavily polluting companies, the impact of air pollution on stock liquidity is greater in non-heavily polluting companies.

### Different industries

The industry attributes of firms may affect the impact of air pollution on the liquidity of individual stocks. The industry classification guidelines for listed companies issued by the China Securities Regulatory Commission (CSRC) in 2012. The industries are specifically classified as follows: A represents agriculture, forestry, animal husbandry and fishery, B represents the mining industry, C stands for manufacturing industry, D represents the production and supply of electricity, heat, gas and water, E stands for construction industry, F means wholesale and retail, G means transportation, storage and postal services, H means accommodation and catering industry, I refers to information transmission, software and information technology services, J represents the financial industry, K represents the real estate industry, L represents leasing and business services, M stands for scientific research and technical services, N refers to water conservancy, environment and public facilities management, O refers to resident services, repair and other services, P refers to education, Q refers to health and social work, R refers to culture, sports and entertainment, S refers to synthesis. Therefore, the sample firms were classified according to industry classification guidelines for listed firms revised by the Securities and Futures Commission in 2012, and the regressions are conducted separately for different industries. To ensure the accuracy of the regression results, industries with <10 listed firms were excluded.

Table 4 presents the regression results of air pollution on stock liquidity for major industries. In general, air pollution has a negative impact on the liquidity of listed firms' stocks. In five industries (manufacturing; construction, transportation, storage and postal services; information transmission, software and information technology services; and real estate). Among them, air pollution has a greater impact on stock liquidity, because their AQI coefficient is larger than that of the other four industries. In the mining and financial industries, air pollution also has a negative impact on stocks liquidity. One possible reason is that the production activities of these industries create more emissions, and changes in air pollution are closely related to this.

**Table 4:** Test for different industries.

**Industry**	**B**	**C**	**D**	**E**	**F**	**G**	**I**	**J**	**K**	**N**
**Variables**	**Turnover**	**Turnover**	**Turnover**	**Turnover**	**Turnover**	**Turnover**	**Turnover**	**Turnover**	**Turnover**	**Turnover**
AQI	−0.0002	−0.0005***	0.0008***	−0.0006***	8.84e−05	−0.0004**	−0.0007**	−9.62e−05	−0.0007***	0.0003
	(0.0002)	(8.65e−05)	(0.0002)	(0.0002)	(0.0002)	(0.0002)	(0.0003)	(0.0001)	(0.0002)	(0.0005)
CSI	0.0727	1.201***	0.196	0.980	0.416	−0.310	4.084***	3.036***	2.006***	0.477
	(0.835)	(0.263)	(0.519)	(0.733)	(0.586)	(0.523)	(1.081)	(0.512)	(0.666)	(1.129)
Annual report announcement date	0.450**	0.647***	0.251*	0.159	0.379**	0.191	0.738***	0.104	0.442**	0.502*
	(0.202)	(0.0639)	(0.132)	(0.191)	(0.149)	(0.139)	(0.259)	(0.130)	(0.175)	(0.284)
Ex–dividend and ex–rights date	−0.0868	−0.0766	−0.141	0.113	0.0404	−0.0322	0.300	0.00981	0.0403	0.0802
	(0.183)	(0.0615)	(0.124)	(0.169)	(0.139)	(0.118)	(0.253)	(0.109)	(0.155)	(0.265)
Month	−0.101***	0.0609***	0.0874***	0.00855	0.132***	0.0657***	0.107***	0.0276*	0.0662***	0.122***
	(0.0272)	(0.0086)	(0.0169)	(0.0239)	(0.0191)	(0.0171)	(0.0352)	(0.0167)	(0.0217)	(0.0369)
Monday	0.0802***	0.0115	0.0167	0.0179	0.0172	0.0249	−0.0148	0.0544***	0.00597	0.0681*
	(0.0263)	(0.00831)	(0.0164)	(0.0232)	(0.0185)	(0.0165)	(0.0341)	(0.0162)	(0.0210)	(0.0356)
Year	Yes	Yes	Yes	Yes	Yes	Yes	Yes	Yes	Yes	Yes
City	Yes	Yes	Yes	Yes	Yes	Yes	Yes	Yes	Yes	Yes
Individual	Yes	Yes	Yes	Yes	Yes	Yes	Yes	Yes	Yes	Yes
Constant term	2.190***	2.210***	0.724***	3.084***	2.429***	1.011***	3.399***	0.768***	5.123***	1.971***
	(0.0548)	(0.0686)	(0.0413)	(0.0455)	(0.0507)	(0.0377)	(0.0919)	(0.0365)	(0.0543)	(0.0670)
Observations	24,197	519,439	41,108	18,175	49,616	31,409	43,568	31,513	43,608	13,284

***, **, *indicate significant at the 1%, 5%, and 10% levels, respectively; Standard errors are shown in parentheses. Same as below; B, C, D, E, F, G, I, J, K and N respectively represent the Mining industry, Manufacturing industry, Electricity, heat, gas and water production and supply industry, Construction industry, Wholesale and retail trade industry, Transportation, storage and postal industry, Information transmission, software and information technology services industry, Financial industry, Real estate industry, Water, environment and public facilities management industry.

### Small cities, medium cities and large cities

The waste generated by residents in their daily lives is one of the major sources of air pollutants. Usually, the larger the city size and the larger the urban population, the more waste is generated by residents' daily life. Therefore, the paper considers whether there is heterogeneity among different city sizes. City size was measured by city population at the end of the year, cities with a population of <500,000 are small cities, between 500,000 and 1,000,000 medium cities, and more than one million are large cities. The full sample is divided into three sub-samples according to the above criteria: small city sample, medium city sample, and large city sample, and regressions were conducted to examine the heterogeneity of different city sizes.

Table 5 examines whether there is heterogeneity across city sizes in the effect of air pollution on stock liquidity based on grouped regressions for samples of different city sizes. The regression results for the small city sample show that the positive relationship between air pollution on stock liquidity is significant at the 1% level. The AQI coefficients of the air quality index are significantly negative at the 10 and 1% levels for the medium and large city samples, respectively. It indicates that city size does affect the negative relationship between air pollution and stock liquidity. Among them, the AQI coefficient in the medium-sized cities is −0.0005, and the AQI coefficient in the large-sized cities is −0.0004, which indicates that the effect estimate in the medium-sized cities is higher. The results in Table 5 indicate that air pollution has a greater impact on stock liquidity in medium-sized cities.

**Table 5:** Test for different city sizes.

**Variables**	**Small**	**Medium–**	**Large**
	**cities**	**sized**	**cities**
		**cities**	
AQI	0.0021***	−0.0005*	−0.0004***
	(0.0007)	(0.000273)	(6.11e−05)
CSI	1.1320	2.3660***	1.1320***
	(1.9290)	(0.8550)	(0.1900)
Annual report announcement date	0.2310	0.7340***	0.5480***
	(0.4740)	(0.2090)	(0.0470)
Ex–dividend and ex–rights date	−0.2670	−0.0794	−0.0390
	(0.5280)	(0.2000)	(0.0442)
Monday effect	0.0149	0.0095	0.0164***
	(0.0610)	(0.0267)	(0.0060)
Month effect	−0.2990***	0.0233	0.0770***
	(0.0629)	(0.0276)	(0.0062)
Constant Term	3.2910***	2.7100***	2.265***
	(0.0734)	(0.0336)	(0.0076)
Year effect	Yes	Yes	Yes
City effect	Yes	Yes	Yes
Individual effects	Yes	Yes	Yes
Observations	15,692	53,622	784,123

***, * show significance at 1%, 10%; Standard errors are in parentheses.

The possible reason for this phenomenon is that although the population of medium-sized cities is less than that of large cities, most of them are located in the central region. The factories originally located in large cities along the eastern coast have been transferred to medium-sized cities in the central region. The exhaust gas generated by these factories causes serious air pollution and has a negative impact on investor sentiment. Finally, pessimism leads to less stock trading activity and poor stock liquidity.

### Mild air pollution and severe air pollution

The air pollution phenomenon is common, and people who have been in a polluted air environment for a long time may become accustomed to it and insensitive to changes in air pollution conditions, which will not trigger changes in investor sentiment and avoid irrational stock trading behavior, i.e., different degrees of air pollution may have different effects on stock liquidity. Therefore, we consider whether there is heterogeneity in different air pollution levels. Taking AQI equal to 300 as the boundary, AQI >300 is classified as heavy air pollution, and <300 as light air pollution. The full sample was divided into two subsamples of light air pollution and heavy air pollution, and regressions were conducted separately.

Table 6 presents the heterogeneity test for different levels of air pollution. We can see that air pollution has a significant effect on stock liquidity at light air pollution levels, the regression results are not significant at heavy air pollution levels. This indicates that in the light air pollution samples, air pollution has a negative impact on stock liquidity.

**Table 6:** Test for different air pollution levels.

**Variables**	**Light air**	**Severe air**
	**pollution**	**pollution**
AQI	−0.0005***	0.0013
	(6.51e−05)	(0.00107)
CSI	1.2350***	−7.195
	(0.187)	(5.195)
Annual report announcement date	0.5550***	0.720
	(0.0461)	(1.679)
Ex–dividend and ex–rights date	−0.0404	0.142
	(0.0435)	(2.048)
Monday effect	0.0159***	0.124
	(0.00591)	(0.199)
Month effect	0.0690***	−0.5160***
	(0.0061)	(0.182)
Constant term	1.1780***	1.7740**
	(0.0623)	(0.860)
Year effect	Y	Y
City effect	Y	Y
Individual effects	Y	Y
Observations	851,238	2,199

***, ** show significance at 1%, 5%; Standard errors are in parentheses.

The possible reason for this phenomenon is a matter of investors' habits. As the phenomenon of air pollution is becoming more common, people become used to air pollution when they are in a severely air polluted environment for a long time; therefore, they do not react promptly to changes in air quality and it does not affect investors' sentiment. However, when people are in a lightly polluted environment, investors are more sensitive to air pollution conditions, and smaller changes in air quality are likely to trigger changes in investor sentiment, thus affecting stock trading behavior and leading to changes in stock liquidity.

### How does air pollution affect stock liquidity?

Table 7 presents the results of air pollution affecting stock liquidity through investor sentiment. Columns (1) and (2) report the empirical results of AQI and investor sentiment (ADL index and ARMS index). The regression coefficients of AQI in Column (1) and Column (2) are −0.5380 and −0.0002 respectively, indicating that when other conditions remain unchanged, each unit of AQI increases, the ADL index decreases by 0.5380 units, and the ARMS index decreases by 0.0002 units. Except for ex dividend and ex dividend date variables, all control variables were significant at the 1% significance level, suggesting that air pollution triggered negative sentiment among investors. Many scholars have confirmed this conclusion, that is, as an environmental factor, air pollution will have a negative impact on investors' mood in the short term, and easily lead to low mood (29, 39–41), worry (42–44), depression (45–48) and anxiety (49, 50).

**Table 7:** Stock liquidity, investor sentiment and AQI.

	**(1)**	**(2)**	**(3)**	**(4)**	**(5)**	**(6)**
**Variables**	**ADL**	**ARMS**	**Sent**	**Turnover rate**		
_AQI_	−0.5380***	−0.0002***	−7.77e−05***	−0.0003***	−0.0004***	−0.0004***
	(0.0233)	(1.17e−05)	(2.01e−05)	(6.01e−05)	(6.01e−05)	(6.00e−05)
_ADL_	–	–	–	3.17e−05***	–	–
				(2.80e−06)		
_ARMS_	–	–	–	–	−0.0242***	–
					(0.0056)	
_Sent_	–	–	–	–	–	−0.0191***
						(0.0032)
_CSI_	78,036***	14.19***	3.0340***	−1.263***	1.5540***	1.2440***
	(72.40)	(0.0363)	(0.0625)	(0.288)	(0.2030)	(0.1870)
Annual report announcement date	−77.20***	0.0344***	0.1230***	0.557***	0.5560***	0.5550***
	(17.86)	(0.0090)	(0.0154)	(0.0462)	(0.0462)	(0.0459)
Ex–dividend and ex–rights date	−8.032	0.0124	0.0413***	−0.0409	−0.0408	−0.0381
	(16.85)	(0.0085)	(0.0145)	(0.0436)	(0.0436)	(0.0434)
Monday effect	184.1***	0.0514***	−0.0466***	0.0101*	0.0171***	0.0150**
	(2.2850)	(0.0012)	(0.0020)	(0.00593)	(0.0059)	(0.0059)
Month effect	69.93***	0.0649***	−0.0337***	0.0646***	0.0684***	0.0694***
	(2.3610)	(0.0012)	(0.0020)	(0.00611)	(0.0061)	(0.0061)
Constant term	87.96***	0.944***	0.2570***	2.322***	2.3470***	2.2980***
	(2.8780)	(0.0014)	(0.0025)	(0.00744)	(0.0091)	(0.0075)
Year effect	Yes	Yes	Yes	Yes	Yes	Yes
City effect	Yes	Yes	Yes	Yes	Yes	Yes
Individual effects	Yes	Yes	Yes	Yes	Yes	Yes
Observations	85,437	853,437	848,576	853,437	853,437	848,576

***, **, * show significance at 1%, 5%, and 10% respectively; Standard errors are in parentheses.

The possible reasons for this phenomenon are as follows: firstly, people are exposed to air pollution and worry about the possibility of suffering from respiratory and cardiovascular diseases in the future, leading to depression. Secondly, more and more toxicological evidence shows that exposure to harmful gases and particulate air pollutants can lead to adverse neurochemical or neuropathological changes, which may manifest or lead to depression, suicidal ideation or related psychological consequences (49, 51–53), and are directly reflected in depression.

Columns (1)–(3) of Table 7 are the regression results of the Equation (4), in which ADL, ARMS and Sent used to measure investor sentiment (InvS). Columns (4)–(6) of Table 7 are the regression results of the Equation (5), in which the investor sentiment (InvS) is measured by ADL, ARMS and Sent in turn. The total effect is the coefficient(β_0_) of AQI in Equation 3. The direct effect is the coefficient(β_6_) of AQI in Equation 5. The intermediate effect is the product of AQI coefficient(β_3_) in Equation 4 and the coefficient(β_7_) of InvS in Equation 5(i.e $\beta_{3}^{*}$ β_7_). The regression coefficient of ADL index in Column (4) of Table 7 is significant at the 1% level. The regression coefficient of AQI is also significant at the level of 1%. The coefficient of ARMS in Column (5) is negative at the 1% level. The arms index is negatively correlated with turnover rate, and AQI coefficient is also significant at the level of 1%. The coefficients of AQI and Sent index in Column 6 are significantly negative at the level of 1%. Combined with the empirical results of Equations (3)–(5), investor sentiment has a partial mediating effect, and investor sentiment is a potential way for air pollution to affect stock liquidity. In particular, when using the ADL index to measure investor sentiment, the total effect is −0.0004, the direct effect is −0.0003, and the intermediary effect is −1.70546e-05(−0.5380^*^3.17e−05). When using ARMS index to measure investor sentiment, the total effect is−0.0004, the direct effect is −0.0004, and the intermediary effect is 0.0484e-05(−0.0002^*^-0.0242). When investor sentiment (InvS) is measured by Sent, the direct effect is−0.0004, and the intermediate effect is 1.48407e-06 (−7.77e-05^*^-0.0191). The above results show that when air pollution is serious, the ADL index is low (the arms index is large or the sent index is low), which leads to more pessimistic investor sentiment and less liquid stocks.

The reason for this phenomenon can be explained using a psychological theory: many psychologists believe that emotion is one of the main driving factors of decision-making. When investors are immersed in negative emotions and face complex trade-offs and choices such as future benefits and costs, they are prone to risk aversion, show a pessimistic tendency, and are unwilling to buy more stocks. Therefore, the stock market is at a low level, and stock trading frequency is reduced, which eventually leads to poor stock liquidity. Considerable literature in investor sentiment also reach this conclusion: air pollution has an adverse impact on the physical and mental health of investors. If investors feel pessimistic, they may not be willing to actively trade (34, 36). Negative sentiment may reduce trading volume (33, 35) and increase insufficient market liquidity (36, 54).

### Robustness test

We use Wu et al. (55) for reference and select Amihud to measure stock liquidity for the robustness test. Table 8 presents the robustness test results. According to Column (4), there is a significant positive correlation between the air quality index and the Amihud index, that is, serious air pollution is often accompanied by the deterioration of stock liquidity. This conclusion is consistent with the above results, indicating that the regression result of the benchmark model is robust.

**Table 8:** Regression results of Amihud on AQI.

**Variables**	**Amihud**	**Amihud**	**Amihud**	**Amihud**
AQI	2.46e−05	2.60e−05*	1.83e−05	2.60e−05*
	(1.51e−05)	(1.51e−05)	(1.50e−05)	(1.51e−05)
CSI	–	−0.713***	−0.750***	−0.7130***
		(0.0470)	(0.0469)	(0.0470)
Annual report announcement date	–	−0.000997	0.00139	−0.0010
		(0.0116)	(0.0116)	(0.0116)
Ex–dividend and ex–rights date	–	−0.000996	0.00162	−0.0010
		(0.0109)	(0.0109)	(0.0109)
Monday effect	–	0.00878***	0.00876***	0.0088***
		(0.00148)	(0.00148)	(0.0015)
Month effect	–	−0.00444***	−0.00468***	−0.0044***
		(0.00153)	(0.00153)	(0.0015)
Constant term	0.0220***	0.0206***	0.0309***	0.0206***
	(0.0018)	(0.00187)	(0.00138)	(0.0019)
Year effect	Yes	Yes	No	Yes
City effect	Yes	No	Yes	Yes
Individual effects	Yes	Yes	Yes	Yes
Observations	853,437	853,437	853,437	853,437

***, * show significance at 1%, 10%; Standard errors are in parentheses.

## Conclusion and implications

Air pollution not only affects people's physical health, but also affects psychological activities and emotions. The pessimism triggered by air pollution affects investors' willingness to buy and hold stocks and damages stock liquidity. Studying the relationship between air pollution and stock liquidity helps to explore the impact of air pollution on the stock market, and has referential value for the relevant authorities in formulating relevant policies. We selects Chinese A-shares from January 1, 2016, to December 31, 2020, as a sample, measured stock liquidity using the index of turnover rate, and measured air pollution in cities using the AQI to empirically analyze the impact of air pollution on stock liquidity. The main findings are as follows: First, the more severe air pollution is, the lower liquid stocks are. Second, investor sentiment is a potential path through which air pollution inhibits stock liquidity, with investor sentiment acting as a partial mediator. Third, heterogeneity analysis finds that the negative relationship between air pollution and stock liquidity varies across heavily and non-heavily polluting firms, different industries, different city sizes, and different degrees of air pollution, with a greater impact in five industries, medium sized cities, light air pollution, and non-heavily polluting firms.

The study provides the following implications based on these findings. Firstly, a sound legal system is an important link in the prevention and control of air pollution. Establish and improve the environmental protection system, establish and improve laws and regulations on the prevention and control of air pollution, establish a reward and punishment mechanism, and urge enterprises to reduce waste emissions; Strengthen environmental protection publicity and education, improve public awareness of environmental protection, realize green production and life, and improve air pollution. Secondly, strict supervision is an important channel to improve information transparency. Improve the environmental protection information disclosure system. Formulate comprehensive disclosure scope and standards, and invite third parties to supervise and audit information disclosure to ensure the authenticity and effectiveness of the disclosed information. Urge enterprises to strengthen ESG information disclosure and widely publicize on multiple platforms to enhance investors' investment confidence, eliminate the negative impact of investors' negative emotions on the stock market and maintain stock liquidity. Thirdly, a real-time monitoring mechanism is an important way to maintain the liquidity of the stock market. Establish investor sentiment monitoring mechanism, investor sentiment evaluation and management mechanism, timely prevent the spread and spread of investors' irrational emotions, and reduce the negative impact of investors' irrational emotions.

## Data availability statement

Publicly available datasets were analyzed in this study. This data can be found at: https://www.gtarsc.com/; http://www.cma.gov.cn/; https://www.cnrds.com.

## Author contributions

CL made great contributions to the design of his works. YY designed, analyzed and interpreted the data, and drafted the first draft. YL, GZ, and LZ put forward suggestions, participated in the adjustment of the project, and contributed to the drafting of subsequent versions of the manuscript. HJ, YX, and YC participated in the review and YY made substantive amendments to it. All authors contributed to the article and approved the submitted version.

## Funding

This work was supported by Sichuan Soft Science Research Project (No.: 2021JDR0165) and University Level Project of Guizhou University of Finance and Economics (No.: 2020XZD01).

## Conflict of interest

The authors declare that the research was conducted in the absence of any commercial or financial relationships that could be construed as a potential conflict of interest.

## Publisher's note

All claims expressed in this article are solely those of the authors and do not necessarily represent those of their affiliated organizations, or those of the publisher, the editors and the reviewers. Any product that may be evaluated in this article, or claim that may be made by its manufacturer, is not guaranteed or endorsed by the publisher.
